# Traffic Signal Control Using Hybrid Action Space Deep Reinforcement Learning

**DOI:** 10.3390/s21072302

**Published:** 2021-03-25

**Authors:** Salah Bouktif, Abderraouf Cheniki, Ali Ouni

**Affiliations:** 1 Department of Computer Science and Software Engineering, University of United Arab Emirates, Al Ain 15551, Abu Dhabi, United Arab Emirates; 2 Department of Electrical Engineering, University of Boumerdes, Boumerdès 35000, Algeria; abderraouf2cheniki@gmail.com; 3 École de Technologie Supérieure, University of Quebec, Montreal, QC H3C 1K3, Canada; ali.ouni@etsmtl.ca

**Keywords:** traffic signal control, traffic optimization, parameterized deep reinforcement learning, P-DQN, hybrid action space

## Abstract

Recent research works on intelligent traffic signal control (TSC) have been mainly focused on leveraging deep reinforcement learning (DRL) due to its proven capability and performance. DRL-based traffic signal control frameworks belong to either discrete or continuous controls. In discrete control, the DRL agent selects the appropriate traffic light phase from a finite set of phases. Whereas in continuous control approach, the agent decides the appropriate duration for each signal phase within a predetermined sequence of phases. Among the existing works, there are no prior approaches that propose a flexible framework combining both discrete and continuous DRL approaches in controlling traffic signal. Thus, our ultimate objective in this paper is to propose an approach capable of deciding simultaneously the proper phase and its associated duration. Our contribution resides in adapting a hybrid Deep Reinforcement Learning that considers at the same time discrete and continuous decisions. Precisely, we customize a Parameterized Deep Q-Networks (P-DQN) architecture that permits a hierarchical decision-making process that primarily decides the traffic light next phases and secondly specifies its the associated timing. The evaluation results of our approach using Simulation of Urban MObility (SUMO) shows its out-performance over the benchmarks. The proposed framework is able to reduce the average queue length of vehicles and the average travel time by 22.20% and 5.78%, respectively, over the alternative DRL-based TSC systems.

## 1. Introduction

Traffic congestion is one of the biggest issues in most of today’s cities causing significant delays and subsequent economic losses [1]. To tackle this issue, several research efforts in the transportation field attempted to develop intelligent transportation systems (ITS) aiming to overcome traffic congestion and improve traffic flow. Traffic signal control systems (TSCs) are one of the key research areas of intelligent transportation systems (ITS) made to control the traffic flow at intersections aiming to reduce traffic congestion [2].

Recently, various research works have leveraged reinforcement learning (RL) to replace the traditional traffic signal control systems [3,4,5]. In contrast with the standard traffic control approaches, RL and Deep RL (DRL) techniques can adapt to diverse traffic situations and conditions. In its recent application to TSC, DRL showed a higher performance over traditional traffic light management techniques [6,7]. In DRL-based traffic light controllers, the objective of the DRL agent is to decide the optimal action which yields improving the TSCs performance. Commonly, the action selection process is based on two strategies. In the first strategy, the DRL agent selects any phase from a finite set of phases without being limited to a predefined sequence of phases [8]. This strategy makes use of the discrete DRL architectures such as Deep Q-Nnetworks (DQN) [9], Double-DQN [10] and Dueling-DQN [11]. However, this strategy lacks the ability to predict the duration of the selected signal phase restricting it from choosing more optimal behavior. Whereas, in the second strategy, the agent’s actions are continuous instead, where the agent decides the duration of the next phase within a predefined cycle of traffic light phases [6]. The latter strategy belongs to the continuous type of DRL algorithms like Deep Deterministic Policy Gradient DDPG [12] and Normalized Advantage Function (NAF) [13]. Unfortunately, these two paths for controlling traffic signals lack flexibility and have not yet used jointly discrete and continuous DRL. Therefore, our ultimate objective, in this paper, is to bridge this gap and propose an approach that takes the potential advantage of combining the two strategies of applying DRL. Our approach is aimed to optimize traffic signal control by deciding simultaneously the proper phase and its associated duration. Hence, we propose a DRL based not only on employing discrete or continuous action spaces exclusively but combines them at the same time. Precisely, being inspired by DRL with parameterized actions [14], our contribution resides in tailoring a Parameterized Deep Q-Networks (P-DQN) architecture [15] that permits a hierarchical decision-making process that primarily decides the traffic light next phases and secondly specifies its associated timing.

This design variant of DRL makes use of a hybrid architecture that combines discrete actions with continuous parameters. Subsequently, the learning agent within the DRL structure chooses at each decision step both the appropriate action and the parameter value associated with that action.

The proposed framework is evaluated by establishing an experimental study that is conducted on the commonly used traffic Simulation of Urban MObility (SUMO) environment. The performance of the proposal as well as the benchmarks are assessed according to the common metrics used for TSC approaches evaluation such as the average travel time, the queue length and the average waiting time of vehicles [8,16]. Remarkably, the evaluation results of our proposed approach show considerable improvements of the TSC performance when compared to the benchmarks.

The rest of this paper is organized as follows. in Section 2, we review the works proposing DRL based solutions for TSC. Section 3 provides preliminaries and theoretical backgrounds needed by hybrid DRL-based TSC solutions. Our approach, as well as the proposed methodology behind it, are described in Section 4. In Section 5, we detail the experimental evaluation of our proposal and discuss the obtained results. Finally, in Section 6, we draw the conclusion and present the potential future works.

## 2. Literature Review

Reinforcement Learning (RL) decision making approach has been widely used in many fields and applications (e.g., transportation, health and energy management) [17,18,19]. In the literature of DRL-based traffic signal control research, transportation engineers and researchers take advantage of deep reinforcement learning to provide optimal TSC systems. Essentially, DRL-based contributions to TSCs focus on improving some of the four main elements of the DRL framework, namely, the state definition, the reward design, the action space and the architecture of the agent. In the following, we narrow the scope of the literature review with a focus on works contributing to improve the action space definition and the architecture of the agent. These works are the closest to our proposal.

### 2.1. Action Space Definitions

The action space embraces the set of possible decisions the agent can take during interaction with the surrounding environment. In DRL-based TSCs, the action space is generally defined according to the preferred way of controlling the traffic lights. First, the action space can be in a binary form where the agent chooses either to maintain the ongoing phase or to skip into the following phase in a predefined sequence of phases [7,16]. The second action space is composed of all possible traffic light phases from which the agent is permissible to select the most appropriate phase at each decision time step [8,20,21]. A third type of action space is rather in the form of a continuous bounded time range, allowing the agent to control the time length of subsequent phases of traffic lights [6,22]. A novel type of action space in DRL-based TSCs encompasses both the discrete and continuous action spaces. This hybrid action space comes in the form of hierarchical discrete-continuous spaces allowing the agent to decides the next phase of the traffic lights and its associated phase timing simultaneously. In this work, beyond the action spaces found in the literature, we will exploit the hybrid action space structure for controlling both the phase selection and timing.

### 2.2. Agent Architecture Specifications

In deep reinforcement learning, the deep neural network is the core element of the agent. The main function of the agent’s network is to learn the optimal policy, mapping input states into optimal output actions. The selection of agent’s network structure depends on the preferred type of action space. When the action space is discrete (e.g., phase selection action type), Deep Q-network (DQN) [9,23] and its extensions (Double-DQN [10], Dueling-DQN [11]) are the popular choices for the agent’s network. The objective of the DQN network is to learn Q-values of actions and decide the optimal action based on the predicted Q-values. On the other side, when the action space is continuous (e.g., phase timing prediction), Policy gradient methods such as Deep Deterministic Policy Gradients (DDPG) [12], Advantage Actor-Critic (A2C) [24] and Soft Actor-Critic (SAC) [25] are most commonly used. However, a more complex action space such as the hybrid action space requires more sophisticated agent’s network structure. Specifically, various architectures have been proposed for the hybrid action structure such as deep reinforcement learning with parameterized action space which is defined as a finite set of discrete actions where each action has an associated continuous parameter value. In the parameterized action space literature, Hausknecht and Stone [26] were first to successfully use deep neural networks in structured (parameterized) action space based on DDPG architecture. Furthermore, Xiong et al. [15] proposed a novel Hybrid framework, known as Parameterized deep Q-network (P-DQN) which comes as a modified version of DDPG architecture showing an improved performance over the previous Hausknecht and Stone framework. On the same trend, Bester et al. [27] fixed some issues found in the P-DQN and proposed a more refined version called as Multi-Pass DQN (MP-DQN). Both P-DQN and MP-DQN structures will be explained and discussed in Section 3 on Backgrounds.

## 3. Background and Preliminaries

In reinforcement learning literature, the problem being tackled is usually formulated as a Markovian Decision Process (MDP) [28], which is characterized by the tuple <S,P,A,R,γ>. The state space is denoted by S, P is the Markov probability of transition, A is the action space, R is the reward and γ is the discount factor. At the time-step *t*, the agent observes the environment state $s_{t}\in S$ and selects an action $a_{t}\in A$ according to its policy π. The agent then receives an immediate reward $R_{t}$ from the environment and observes the next state $s_{t+1}∼P\left(s_{t+1}|s_{t},a_{t}\right)$. The agent’s policy π can be either stochastic or deterministic. When the policy π is deterministic, π(a|s) maps each state $s_{t}$ to a specific action $a_{t}$. Whereas in the stochastic policy, π(a|s) maps each state to a probability distribution over the action space A. The agent’s goal is to derive a policy π which maximizes the cumulative discounted reward $G_{t}=\sum_{k=0}^{n}{\gamma^{k}R}_{t+k}$ starting from the time-step *t* [28].

### RL for Hybrid Action Space

A common type of action space in real-life applications consists of both discrete and continuous action spaces (hybrid action space in short). A related work in hybrid action space literature includes the parameterized action space, which is defined as a finite set of actions, where each action is parameterized by a continuous value [15]. We consider formulating our decision problem as a Markovian Decision Process with a parameterized hybrid action space A as in the proposed P-DQN architecture by Xiang et al. [15]. The action space is defined as:

(1) $$A=\{\left(k,x_{k}\right)|x_{k}\in X_{k}\;\mathrm{for}\;\mathrm{all}\;k\in \left[K\right]\},$$

where $\left(k,x_{k}\right)$ is a joint action in the action space A that follows a hierarchical structure when choosing an action. Hence, we have a primary action *k* chosen from a discrete set *K* (k∈K={1,...,K}), and a subaction consists in determining a continuous parameter $x_{k}\in X_{k}$ from a continuous action space. The action space $X_{k}$ defines the domain of the parameters associated with primary actions *k*. Given the new action space, the *Q*-value function is denoted $Q(s,k,x_{k})$ instead of Q(s,a), where s∈S, a∈A, k∈[K] and $x_{k}\in X_{k}$. Therefore, at the time-step *t*, The Bellman equation of *Q*-function is given as:

(2) $$Q\left(s_{t},k_{t},x_{k_{t}}\right)\;=E_{s_{t+1}}\left[R_{t}+\gamma \max_{k\in [K]}\underset{x_{k}\in X_{k}}{\sup}Q\left(s_{t+1},k,x_{k}\right)|s,k,x_{k}\right].$$

Like in the DDPG [12], the $x_{k}^{*}=\mathrm{argsup}\underset{x_{k}\in X_{k}}{Q}\left(s_{t+1},k,x_{k}\right)$ can be viewed as a function $x_{k}^{Q}:S\to X_{k}$, mapping the state space to the continuous domain of action parameters. Consequently, two mappings are needed to select the action and its parameter; the *Q*-function becomes:

(3) $$Q\left(s_{t},k_{t},x_{k_{t}}\right)\;=E_{s_{t+1}}\left[R_{t}+\gamma \max_{k\in [K]}Q\left(s_{t+1},k,x_{k}^{Q}\left(s_{t+1}\right)\right)|s_{t}=s\right].$$

Similarly to DQN and DDPG, both discrete and continuous mappings take advantage of deep neural networks to approximate the $Q(s,k,x_{k})$ and $x_{k}^{Q}$ mappings. In particular, $Q(s,k,x_{k};\omega )$ with network weights ω approximates $Q(s,k,x_{k})$ and $x_{k}\left(\cdot ;\theta \right)$ with network weights θ approximates $x_{k}^{Q}$ mapping. Similar to *Q*-learning, the target $y_{t}$ is defined as:

(4) $$y_{t}=R_{t}+\gamma \max_{k\in [K]}Q\left(s_{t+1},k,x_{k}\left(s_{t+1};\theta_{t}\right);\omega_{t}\right),$$

The loss functions for updating the parameters ω and for updating the θ are respectively defined as:

(5) $$\ell_{t}^{Q}\left(\omega \right)=\frac{1}{2}\left[Q\left(s_{t},k,x_{k}\right);\omega_{t}\right)-y_{t}{]}^{2},\;\mathrm{and}$$

$$\ell_{t}^{\Theta }\left(\theta \right)=-\sum_{k=1}^{K}Q\left(s_{t},k,x_{k}\left(s_{t};\theta \right);\omega_{t}\right)$$

**Figure 1:**
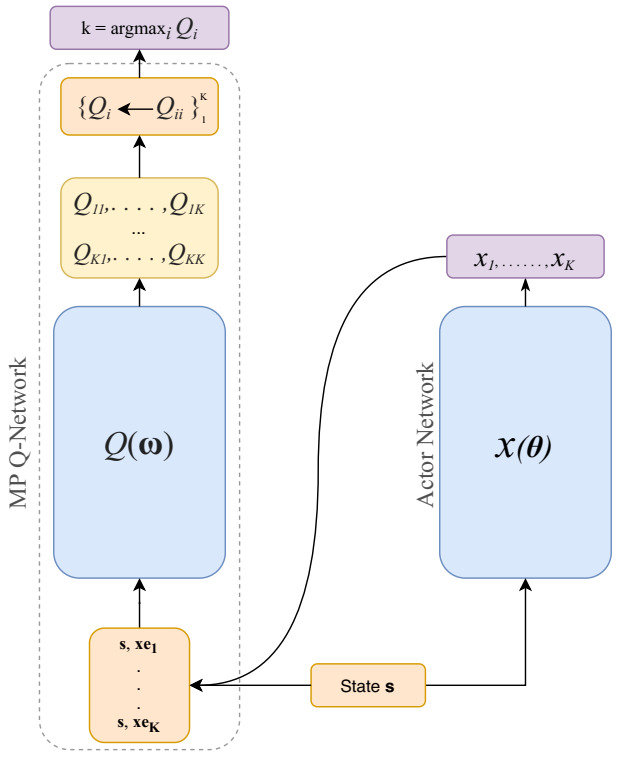
Structure of MP-DQN architecture.

One issue the P-DQN architecture suffers from is the the joint action-parameters input to the *Q*-network, where each *Q*-value of an action *k* depends not only its associated action-parameter $x_{k}$ but instead all the action-parameters $\left(x_{1},\cdots ,x_{K}\right)$ are engaged. This invalidates the P-DQN theoretical foundations claimed by Xiang et al. [15]. As a solution to this issue, Bester et al. [27] proposed a modified variant to P-DQN, namely, Multi-Pass DQN (MP-DQN), by separating each $x_{k}$ action-parameter with its associated action *k*. The MP-DQN involves performing multiple forward passes to the network, once per action *k*, with the state *s* and action-parameter vector ${\mathrm{xe}}_{k}$ as input to the MP Q-Network (see Figure 1). Notice that the vector ${\mathrm{xe}}_{k}=\left(0,\cdots ,0,x_{k},0,\cdots ,0\right)$ is the standard basis for dimension *k*. Introducing the vector ${\mathrm{xe}}_{k}$ solves the P-DQN issue and makes $Q_{k}$ dependent only on the associated $x_{k}$ where:

(6) $$Q\left(s,k,{\mathrm{xe}}_{k}\right)≊Q\left(s,k,x_{k}\right).$$

## 4. Parameterized Deep Reinforcement Learning Approach for TSC

In this section, we discuss the proposed approach to control the traffic signals using a specific reinforcement learning framework called Parameterized-DQN to generate both the appropriate phase P of the traffic signals and its corresponding duration $d_{P}$.

**Figure 2:**
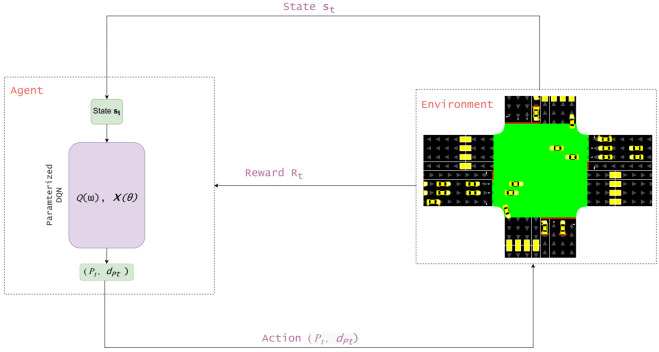
Overview of the framework structure for traffic signal control with Phase and Duration control.

Our framework is depicted in Figure 2 showing the overall structure of our proposal. At every time-step *t*, the current state $s_{t}$ of the intersection environment represented as a vector is being observed by the learning agent. Then, the latter maps the state vector to actions π:S→A, using its latest policy π: of controlling the TSC. The joint action $a_{t}=\left(P_{t},d_{P_{t}}\right)\in A$ consists of selecting the primary action, the appropriate phase P, and at the same time its associated subaction (i.e., the phase duration $d_{P}$). As a result of applying these actions at two levels of the traffic light settings, the agent receives, from the environment, a reward $R_{t}$ as well as the next state $s_{t+1}$ after a lapse of time $t_{P}$ proportional to $d_{P}$. The resulting experience is stored at every time-step as a tuple $<s_{t},a_{t}=\left(P_{t},{d_{P}}_{t}\right),R_{t},s_{t+1}>$ in the agent’s memory *M* for further replay during the agent’s training process. By considering a Parameterized MDP setting, some RL elements should be defined, namely, the state space, the action space, the reward function and the agent architecture.

### 4.1. State Space

**Figure 3:**
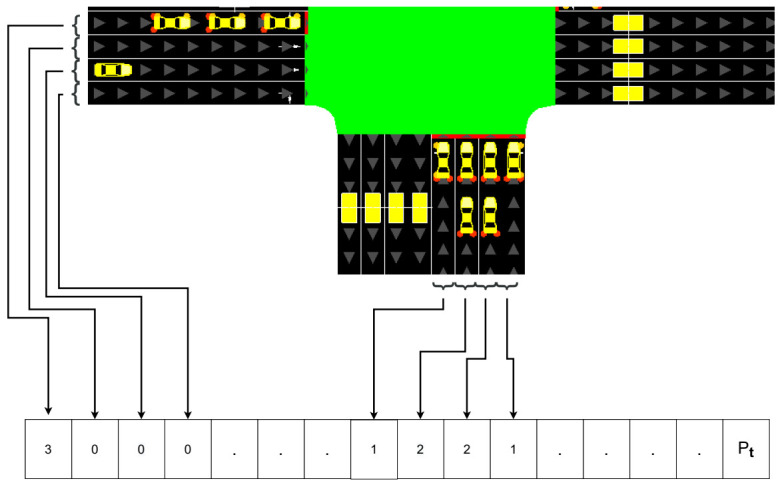
Example of the state vector extracted from the intersection environment.

For an intersection environment, we define the state vector $s_{t}$ as the queue length of vehicles $q_{l}$ in each lane *l* at time step *t*, in addition to the current phase of signals $P_{t}$. Queuing vehicles in the environment are those vehicles with speed less than 0.1 m/s during the simulation. We consider the total number of lanes L=16, in addition to the current phase of signals $P_{t}$ represented by an integer in {0,1,2,3}. Figure 3 shows an example of the acquired vector state from a real time traffic intersection.

Let **$s_{t}$** be the state vector at a given time step *t* where **$s_{t}$**$\in R^{L+|P|}$, and |P|=1 is the dimension of the phase vector. Thus, the vector $s_{t}$ is formulated as:

(7) $$s_{t}\left(q\right)=\left[\begin{array}{c} {q_{0}}_{t}\\ {q_{1}}_{t}\\ .\\ .\\ {q_{L-1}}_{t}\\ P_{t} \end{array}\right]$$

### 4.2. Reward Function

The reward function r:S×A→R maps the joint-actions $a_{t}=\left(P,d_{P}\right)\in A$ and states $s_{t}\in S$ into a scalar value $R_{t}\in R$. The immediate reward $R_{t}$ evaluates how good the taken joint-action $a_{t}$ in the current state $s_{t}$ is. Along with the above state definition, we define the reward function as the negative sum of queuing vehicles at time-step *t* stated as follows:

(8) $$R_{t}=-\sum_{l=0}^{L-1}q_{l}=-\sum_{l=0}^{L-1}s_{t+1}\left(q\right)$$

### 4.3. Action Space

**Figure 4:**
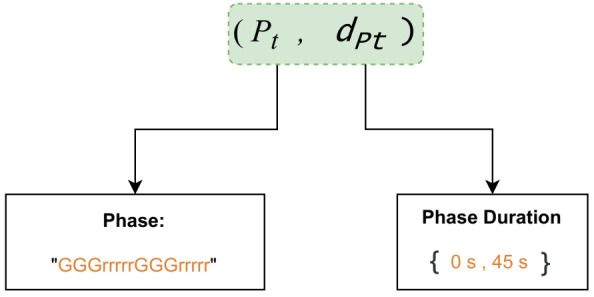
Example of the agent’s action that is applied to the traffic light.

The most important part of this setup involves controlling the traffic signals’ behavior by selecting the appropriate actions at each time-step. The action space is built-up of two hierarchical subspaces, respectively, of traffic light phases and the associated phase durations. Therefore, an action $a=(P,d_{P})$ is a joint action with a hierarchical structure, where *P* is the primary action which indicates a phase of the traffic signals and $d_{P}$ is the secondary parameter indicating the duration of the phase *P*. In this work, we define the first subspace as a set of four phases ∈{0,1,2,3} and the second subspace as the domain of phase duration, a continuous time interval, where $d_{P}\in \left[t_{min},t_{max}\right]$. Thus, the action space is defined as $A=\{\left\{0,1,2,3\right\}\cup \left\{\left[t_{min},t_{max}\right]\right\}\}$. An example of the joint action is illustrated in Figure 4 where the phase *P* constitutes a set of nonconflicting signals ("G" for green, "r" for red and "y" for yellow) to control each traffic movement, and the duration $d_{P}$ falls in the interval [0 s, 45 s].

### 4.4. Agent’s Architecture

**Figure 5:**
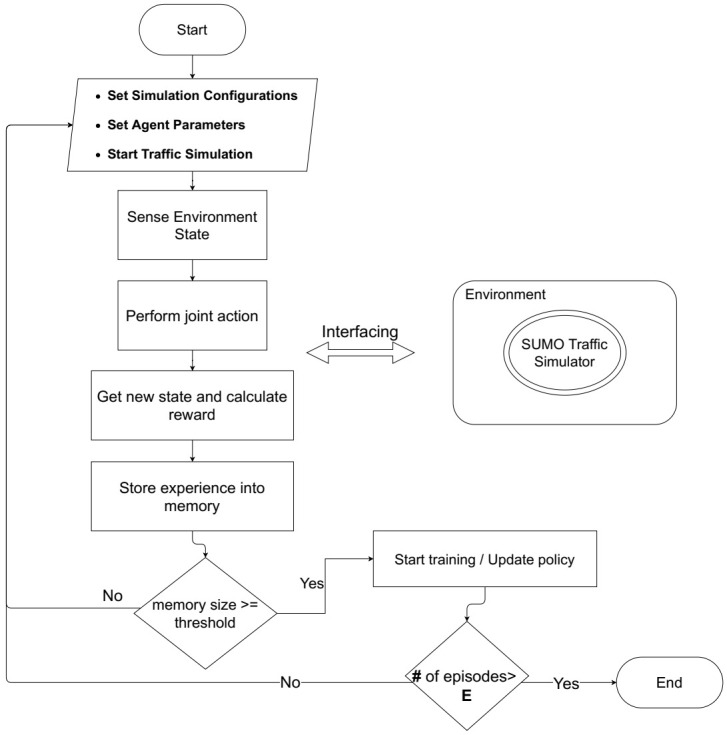
Flow diagram describing the dynamic of the proposed framework.

Following the hybrid nature of the defined action space above, most appropriate architectures that fit our proposal fall in the family of reinforcement learning with parameterized action space architectures (e.g., Paramterized Q-Network [15], Multi-Pass Q-Networks [27]). Multi-Pass-DQN is notably a well performing agent’s architecture that has been proposed recently by Bester C. et al. [27] as a modified version of the P-DQN made to deal with hierarchical hybrid action spaces. In MP-DQN as adopted in our approach, two neural networks are employed, one for approximating the value based *Q*-function to select the high-level discrete action *P* denoted by $Q(s,P,d_{P};\omega )$, we call it the Actor network. The second network is used to approximate the policy based mapping ${x_{d}}_{P}$ to predict the low-level continuous duration, denoted as ${x_{d}}_{P}\left(s;\theta \right)$, we call it the ParamActor network. For stability purpose, both networks are accompanied with target networks that are used in predicting target values $y_{t}$ and updating the main networks. The architecture of the Actor neural network Q(ω) is composed of an input layer of size 16+1+4 (where 4 is the number of action-parameters), a hidden layer of 256 neurons with Relu activation function, and an output of size 4 to approximate the *Q*-values of discrete actions. For the network architecture of the ParamActor x(θ), we use an input layer of size 16+1, a hidden layer of 256 neurons with Relu activation function and an output of size 4 neurons to predict the continuous action-parameters associated with the discrete Actor actions. Figure 5 illustrates a dynamic flow of the proposed framework composed of mainly five iterative processes. By setting up the simulation configurations and the learning parameters, the agent iteratively perceives traffic state, performs the joint action and observes the new traffic condition along with the obtained reward. These processes are stored in the agent’s memory. The update of the agent’s policy starts when the content of the memory exceeds a certain threshold, and continue, in every time step until a termination condition (i.e., reaching a Maximum number of episodes *E*). Algorithm 1 as well provides a pseudocode of the training operation of the proposed framework, Traffic Signal Control Using Parameterized Deep RL. Initially, the essential parameters ($\left\{lr_{Q},lr_{x}\right\},\epsilon ,B,\zeta ,\omega_{0},\theta_{0}$) are initialized to begin the simulation and the training operation of the framework. For a range of *E* episodes and for each time-step *t* in every episode, the agent observes the traffic state $s_{t}$ and selects a joint action $a_{t}=\left(P_{t},{d_{P}}_{t}\right)$ according to ϵ-greedy policy,

(9) $$a_{t}=\left\{\begin{array}{c} a\;\mathrm{sample}\;\mathrm{from}\;\;\zeta \;\;\;\mathrm{with}\;\mathrm{probability}\;\epsilon ,\\ \left(P_{t},{d_{P}}_{t}\right)\;P_{t}=\underset{P}{\mathrm{argmax}}Q\left(s_{t},P,{d_{P}}_{t};\omega_{t}\right)\;1-\epsilon , \end{array}\right.$$

where ζ is a uniform random distribution over a bounded continuous interval $\left[t_{min},t_{max}\right]$. The joint action $a_{t}=\left(P_{t},d_{P_{t}}\right)$ is applied to the traffic signal settings and the resulting traffic state $s_{t+1}$ is obtained as well as the rewarding signal $R_{t}$. Each resulting experience i.e., $<s_{t},\left(P_{t},d_{P_{t}}\right),R_{t},s_{t+1}>$ is stored in a memory *M* for further replay. After collecting an enough number of experiences exceeding the initial memory threshold, a random batch of size *B* is sampled from the memory to compute target value $y_{t}$. The target value $y_{t}$, state $s_{t}$ and action $a_{t}$ are used to calculate the gradients $\nabla_{\omega }\ell_{t}^{Q}\left(\omega_{t}\right)\;\mathrm{and}\;\nabla_{\theta }\ell_{t}^{Q}\left(\theta \right)$ which in turn are utilized besides the learning rates $\left\{lr_{Q},lr_{x}\right\}$ to update ω and θ weights.
**Algorithm 1** Traffic Signal Control Using Parameterized Deep RL.1:Initialize: Learning rates $\left\{lr_{Q},lr_{x}\right\}$, exploration parameter ϵ, minibatch size *B*, a probability distribution ζ, flow configurations, network weights $\omega_{0}$ and $\theta_{0}$.2:**for** episode e=1,⋯E **do**3:    Start simulation, observe initial state $s_{0}$ and take initial joint action $a_{0}$.4:    
**for** t=1,⋯T **do**
5:         Compute action parameters ${d_{P}}_{t}\leftarrow {x_{d}}_{P}\left(s_{t};\theta_{t}\right)$.6:         Select action $a_{t}=\left(P_{t},{d_{P}}_{t}\right)$ according to ϵ-greedy policy.
$$a_{t}=\left\{\begin{array}{c} a\;\mathrm{sample}\;\mathrm{from}\;\;\zeta \;\;\;\mathrm{with}\;\mathrm{probability}\;\epsilon ,\\ \left(P_{t},{d_{P}}_{t}\right)\;P_{t}=\underset{P}{\mathrm{argmax}}Q\left(s_{t},P,{d_{P}}_{t};\omega_{t}\right)\;1-\epsilon . \end{array}\right.$$7:         Perform $a_{t}$, observe next state $s_{t+1}$ and get $R_{t}$.8:         Store $<s_{t},a_{t},R_{t},s_{t+1}>$ in memory *M*.9:         Sample random *B* experiences from *M*.10:$$y_{t}=\left\{\begin{array}{c} R_{t}\;\;\;\mathrm{if}\;\;t=T,\\ R_{t}+\max_{P}\gamma Q\left(s_{t+1},P,{x_{d}}_{P}\left(s_{t+1};\theta \right);\omega_{t}\right)\;\mathrm{otherwise}. \end{array}\right.$$11:         $\mathrm{Compute}\;\nabla_{\omega }\ell_{t}^{Q}\left(\omega_{t}\right)\;\mathrm{and}\;\nabla_{\theta }\ell_{t}^{Q}\left(\theta \right)\;\mathrm{using}\;\left\{y_{t},s_{t},a_{t}\right\}$.12:         update weights $\omega_{t+1}\overset{}{\leftarrow }\omega_{t}-lr_{Q}\nabla_{\omega }\ell_{t}^{Q}\left(\omega_{t}\right)$ and $\theta_{t+1}\overset{}{\leftarrow }\theta_{t}-lr_{x}\nabla_{\theta }\ell_{t}^{Q}\left(\theta \right)$.13:    
**end for**
14:**end for**

## 5. Experiments

In this section, we present our empirical study to evaluate our proposed framework using simulation based traffic. We first present the experiment setup, parametric settings and the performance evaluation metrics. Then, we describe a set of baseline approaches which serve as benchmarks for comparison. Finally, we present and discuss the simulation results of the proposed approach considering different scenarios and performances of benchmarks.

### 5.1. Experiment Setup

In our experimental study, we utilize the open source Simulation of Urban MObility (SUMO) simulator [29] to simulate the intersection environment and traffic. SUMO has been widely used in several recent works [5,7,30] and provides an API package called TraCI to interface with Python programming language. For the agent architecture, we adopt and customize the implementation of the MP-DQN inspired by Bester et al. [27] which is available online (https://github.com/cycraig/MP-DQN, accessed on 22 August 2020).

We consider a typical 4-way geometry (i.e., East, West, North, South) for the structure of the intersection with each incoming/outgoing road having 4 lanes. All the lanes are of 750 m length with maximum lane speed set to 13.89 m/s (i.e., the urban areas speed limit [31]). They also have the same priority and the same width. The left-most lane is dedicated for turning left solely and the rest of lanes can be occupied by straight or right-turn vehicle-movements. A yellow phase follows the green phase and lasts 3 s for safety reasons.

The traffic flow simulation is generated using custom scripts to simulate realistic traffic flows. In particular, the traffic begins with a low number of cars, increases during the rush hours to its peak value, then, it decays back to a low number of vehicles. We simulate such a scenario (i.e., low, high and low) for a time window of three hours (10,800 s) by having approximately one hour of nonuniform low flow followed by a second hour of nonuniform high flow, then a third hour of nonuniform low flow. Each vehicle of the flow has an Origin point (*O*) and a Destination point (*D*) and follows the route OD (i.e., from *O* to *D*). The vehicle routes include going straight movements (North-South and East-West) and turning movements (Left-turns and Right-turns). We set the traffic generation in a way to estimate that 75% of the vehicles are moving straight and 25% are turning right or left. Detailed about simulated traffic flow are presented in Table 1.

### 5.2. Parameters and Training Settings

A number of parameters need to be set to leverage the performance of our proposed approach. After several runs within different scenarios, the adopted hybrid architecture has been tuned and its parameters are empirically determined. In particular, the number of training episodes *N* is set to 301 with an episode duration of 3800 s (extra 200 s to free up the late inserted vehicles to the simulation). Our agent is set in order to follow ϵ−greedy discrete policy with random uniform continuous action selection. Such a policy is shown empirically to perform better than the common Ornstein–Uhlenbeck noise (which was recommended by the original DDPG’ authors for the sake of action exploration). The exploration parameter ϵ is decreased linearly from 1 to 0.01 during 270 episodes where the agent explores new actions. The size of the replay memory is set to 20,000. Our agent starts learning and updating its policy when the memory content reaches 128 experiences (Memory Training threshold, called Tr_threshold). At every time step, the training set for the agent is a mini-batch of b=64 experiences, where an experience is a tuple of $<s_{t},a_{t}=\left(P_{t},{d_{P}}_{t}\right),R_{t},s_{t+1}>$. Our set of parameters also includes those of the agent learning networks, where the number of nodes in hidden layer is set to 256, and RMSProp [32] stochastic gradient decent method is used for updating both Actor and ParamActor networks weights with a learning rate of $lr_{Q}=0.001$ and $lr_{x}=0.00001$ respectively. While γ discounting factor, set to 0.95, is used for updating the *Q*-values. A gradient clipping method for the gradients is applied with a value of 1 which accelerates the training of the networks. Equally, as suggested by Hausknecht and Stone [26], the inverting gradients method is used to keep the action parameters in their bounded region. Table 2 summarizes various parameters used with their associated values.

### 5.3. Performance Evaluation Metrics

Following the literature, traffic signal control approaches are evaluated using three main metrics [8,16], (1) Average Travel Time (ATT), (2) the queue length (QL) and (3) the average waiting time of vehicles (AWT).

#### 5.3.1. Average Travel Time (ATT).

It is defined as the total travel time of all vehicles divided by the number of vehicles, formally expressed by the following equation:

(10) $$ATT=\frac{1}{N_{veh}}\sum_{j=0}^{N_{veh}}t_{j,start}-t_{j,end}$$

where $N_{veh}$ is the total number of vehicles, $t_{j\_start}$ is the time the vehicle *j* enters the environment and $t_{j\_end}$ is the time the vehicle *j* exited the environment.

#### 5.3.2. Average Waiting Time (AWT).

This metric corresponds to the average waiting time spent by the vehicles. A vehicle is considered as waiting if its speed is less than 0.1 m/s since the last time it was faster than 0.1 m/s, otherwise the waiting time counter is reset to 0 (i.e., as it starts moving with a speed >0.1 m/s). The formula for the average waiting time is given by:

(11) $$AWT=\frac{1}{N_{veh}}\sum_{j=0}^{N_{veh}}WT_{j},$$

where $WT_{j}$ is the total waiting time of a vehicle *j* during an episode.

#### 5.3.3. Queue Length (QL).

The queue length of a lane is the total number of vehicles queuing on a lane. The queuing vehicles are those with a speed less than 0.1 m/s on the given lane (known in SUMO as vehicle in ‘halting’ state). We consider the sum of queues lengths over all lanes:

(12) $$QL=\sum_{l=0}^{L}q_{l},$$

where *L* is the total number of lanes and $q_{l}$ is the queue length on lane *l*.

### 5.4. Benchmarks

To evaluate of the performance of the proposed framework, we compare it to the traditional Fixed-Time as well as the DQN discrete action space approach and the continuous action space DDPG approach.

#### 5.4.1. Fixed Time Approach

It is the simplest traffic control approach that uses fixed phase duration with fixed cycle length and fixed order [33]. The duration of green phases is set to 30 s and the yellow phase duration is 3 s.

#### 5.4.2. Discrete Approach

There are several proposals in the literature which use the deep reinforcement learning DQN approach for traffic signal control [5,7,8]. The DQN agent predicts solely the next phase from a set of phases with a fixed phase duration. We consider the implementation which makes use of the Double-DQN (DDQN) with Prioritized Experience Replay Memory (PER) as the agent’s architecture. For the state and reward definitions we use the queue length for both the state and reward formulae.

#### 5.4.3. Continuous Approach

This approach takes advantage of the continuous DRL architecture to control the traffic signals [6]. It only predicts the duration of the next phase where the sequence of the phases is kept fixed. We use the DDPG continuous architecture for the agent. The state and reward are defined as the latter discrete based approach.

### 5.5. Results and Discussion

**Figure 6:**
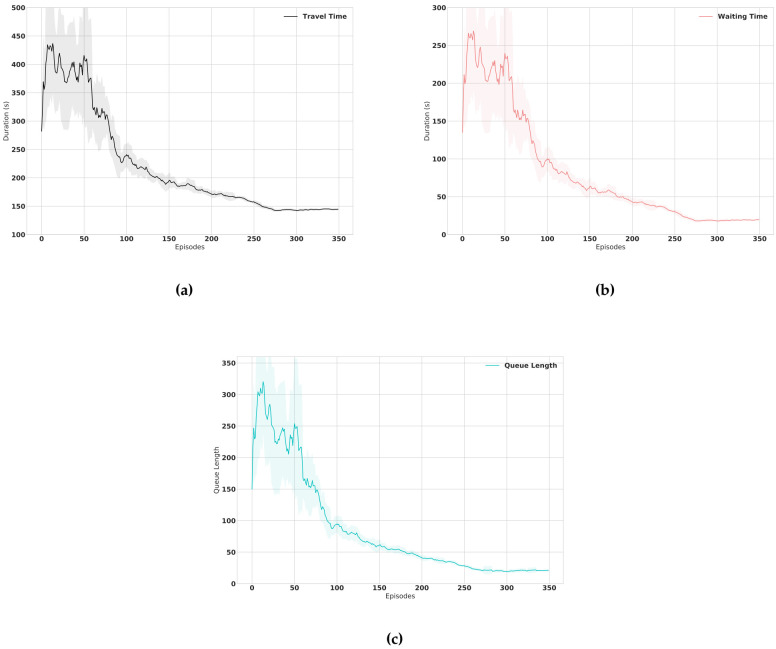
Learning curves of the proposed framework for (**a**) Average Travel Time, (**b**) Average Waiting Time and (**c**) Average Queue Length over episodes.

We train the agent on the simulation setup using the training parameters discussed earlier. The resulting smoothed training curves of the proposed framework are illustrated in the Figure 6. It can be noticed from the learning curves that the training undergoes what is known as a “cold start” [34] problem at early stages due to the exploration of the unfamiliar environment where the agent randomly applies decision actions. The agent subsequently optimizes its performance after grasping enough experience batches.

**Figure 7:**
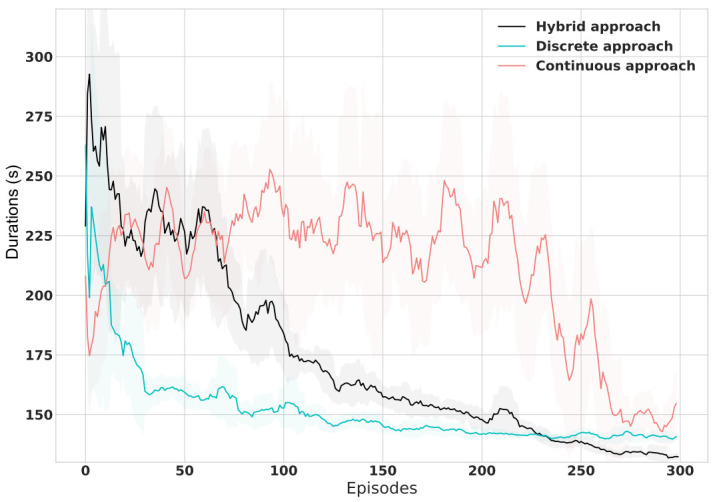
Travel Time Training Curves Comparison of Hybrid Framework Against Discrete and Continuous Benchmarks.

Figure 7 shows the learning performance comparison against the Discrete and Continuous baselines. Remarkably, the Discrete approach exhibits fast initial learning but plateaus at lower performance than the Hybrid framework. It initially learns faster due to the fact that it already has a fixed phase timing and needs only to select the more suitable phase. The Continuous approach curve swings until it reaches a better performance but still worse than the rest. On the other hand, the Hybrid approach curve exhibits a linear-like decaying until it crosses the baselines’ curves where it outperforms the benchmarks’ performance.

**Figure 8:**
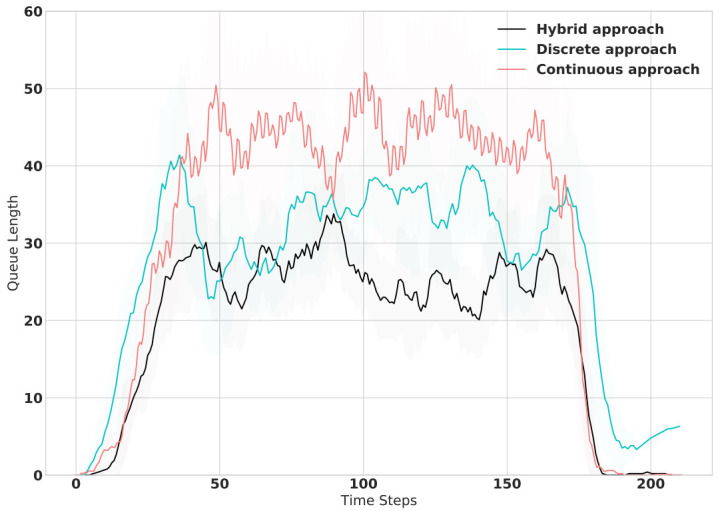
Queue Length performance comparison of Hybrid approach versus Discrete and Continuous baselines during traffic simulation.

In Table 3, we observe the average travel time scores of the Fixed-Time, Discrete and Continuous benchmarks versus the proposed framework with C1-C6 are the simulation configurations listed in Table 1. Notably, the Fixed Time approach is far behind the other approaches due to its static behavior as opposed to the dynamic characteristics of the traffic flow. On the other side, one can remark that the deep reinforcement learning frameworks show noticeable results as they are more capable of dealing with dynamic conditions. Out of the DRL approaches, the proposed Hybrid actions framework outperforms the rest of the approaches in all simulated experiments. This is due to the fact that the Hybrid framework controls the TSC more flexibly by selecting the appropriate phase as well as its duration simultaneously. Further evaluations are shown in Figure 8 where we compare the queue length performance of the three deep RL approaches during one simulation episode. Similar to travel time performance results, the performance of the proposed approach surpasses the baselines by keeping the queue length lower throughout the traffic simulation.

## 6. Conclusions and Future Work

In this work, we have addressed the traffic signal control dual problem involving next phase determination and its duration. We aim to solve such a problem by leveraging the state of the art of a hybrid reinforcement learning variant. Specifically, we tailor the hybrid parameterized Deep Q-Networks, namely, Multi-Pass DQN, to dually control the TSC phase and its associated timing jointly. We conducted a simulation that allowed a series of controlled experiments for evaluating and demonstrating our framework performance. Moreover and for the sake of validity, we compared our framework to Deep RL benchmarks during training and taking decision at the intersection. The evaluation of the performance of our approach made use of the average travel time and the vehicle queue length as practical metrics. The results proved that our hybrid DRL variant outperformed the baselines in all the simulated experiments. A significant reduction of the average queue length of vehicles and the average travel time by 22.20% and 5.78%, respectively. The potential advantage of our framework is its hybrid nature, which allowed the TSC to control the phase selection as well as its duration. Our future works are twofold. Indeed, we would like to extend the scope of our hybrid DRL in order to cover more than one intersection in different ways, e.g., centralized and decentralized. In the second extension, we will direct our further simulations and experiments using real data from real world traffic intersections.

## Figures and Tables

**Table 1:** Simulated traffic flow.

Distribution Type	Configuration	Generated Flow (Vehicles)	Start Time (s)	End Time (s)
Weibull Dist	C1 C2 C3	1500 4000 C1-C2-C1	0 s 0 s 0 s	3800 s 3800 s 11,000 s
Normal Dist	C4 C5 C6	1500 4000 C4-C5-C4	0 s 0 s 0 s	3800 s 3800 s 11,000 s

**Table 2:** Parameters Setting for Agent Training.

Parameter	Description	Value
*N*	*Number of training episodes*	301
*M*	*Replay Memory*	20,000
Tr_threshold	*Memory Training threshold*	128
*b*	*Mini-batch size*	64
$lr_{Q}$	*Actor Learning rate*	0.001
$lr_{x}$	*ParamActor Learning rate*	0.00001
γ	*Gamma factor*	0.95
eps_min	*minimum value of epsilon*	0.01
epsilonepisodes	*Number of epsilon episodes*	270
yellowduration	*Yellow phase duration*	3 s

**Table 3:** Performance comparison of our framework to others with respect to average travel time (s).

	C1	C2	C3	C4	C5	C6
Fixed-Time	164.94	254.01	217.10	165.45	255.64	223.92
Discrete approach	142.07	149.5	147.28	139.10	147.86	145.97
Continuous approach	148.46	167.02	160.95	137.03	160.63	154.25
**Hybrid approach**	**133.61**	**141.05**	**138.33**	**130.59**	**138.98**	**136.89**

## Data Availability

Synthetic Data and Codes that support the findings of this study are openly available at https://github.com/abderraouf2che/Hybrid-Deep-RL-Traffic-Signal-Control, accessed on 22 August 2020.
